# Electric-Field-Induced Tautomerism in Metal-Free Benziporphyrins Enables Aromaticity-Controlled Conductance Switching

**DOI:** 10.1021/acs.nanolett.5c06447

**Published:** 2026-04-15

**Authors:** Yenni Ortiz-Acero, Arnau Cortés-Llamas, Jordi Ribas-Arino, Stefan T. Bromley

**Affiliations:** † Departament de Ciència de Materials i Química Física & Institut de Química Teòrica i Computacional (IQTCUB), 16724 Universitat de Barcelona, c/Martí i Franquès 1-11, 08028 Barcelona, Spain; ‡ Institució Catalana de Recerca i Estudis Avançats (ICREA), Passeig Lluís Companys 23, 08010 Barcelona, Spain

**Keywords:** metal-free porphyrins, tautomerism, single-molecule junctions, electric-field gating, molecular switches, quantum transport

## Abstract

Metal-free porphyrins can alternate between hydrogen-bonded tautomers, potentially enabling reversible molecular switching. However, controlled electric-field gating of porphyrin tautomerism has not been fully realized. We propose metal-free benziporphyrins (MFBPs), in which one pyrrole ring is replaced with a phenol group, as a new platform for tautomer-based molecular electronics. This approach allows for three distinct tautomers, each possessing a characteristic aromatic or antiaromatic electronic structure. Density functional theory and quantum transport calculations show that (i) experimentally realizable electric fields can induce selective tautomerization and (ii) each tautomer exhibits a characteristic conductance profile. The strong switching capability of MFBPs is demonstrated by on/off ratios exceeding 500 at low bias. Fused MFBPs further expand the functionality by providing multiple tautomeric states for multistate molecular registers and high-conductance wire-like architectures. These results establish MFBPs as versatile building blocks for electric-field-responsive molecular devices and open new research opportunities for molecular-scale logic and memory.

## Graphical abstract

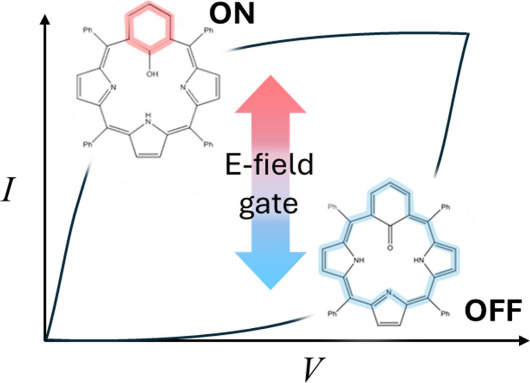

Porphyrins have attracted a considerable amount of attention in molecular device research due to their chemical stability, extended π-conjugation, and ease of functionalization.^1,2^ Their four inner nitrogen atoms readily coordinate metals, enabling the precise control of electronic and optical properties in molecular devices^3,4^ and functional materials.^5^ In metal-free porphyrins (MFPs), two hydrogen atoms occupy the inner nitrogen sites. These hydrogens undergo rapid tautomerism, effectively transferring as protons, while preserving the neutral, fully conjugated π-electron system of the macrocycle. This dynamic behavior has minimal impact on the delocalized π-electron magnetism, making MFPs promising candidates for molecular spintronic applications.^6,7^ However, tautomerism can locally modulate electronic properties, enabling MFPs to serve as platforms for molecular switching via controlled proton transfer.^8–11^ Such tautomeric switching is particularly appealing for device applications because it (i) generates well-defined, distinct molecular states, (ii) is reversible, and (iii) occurs without significant structural perturbation of the porphyrin macrocycle.

For practical device switching, the tautomers in MFPs must be accessible via external stimuli. This control was first demonstrated by varying the current through single MFPs adsorbed on a surface,^8^ and conductance-based tautomerism has also been observed in single-molecule junction experiments.^11^ These studies, together with theoretical work,^9^ show that different tautomeric structures exhibit distinct electronic transport properties. For device applications, electric-field (E-field) gating is highly desirable. Previous work on the effects of E-fields on molecular structure in junctions has shown that applied gating fields can twist dipolar phenyl rings.^12^ This conformational response has been invoked in theoretical predictions of gate-controlled conductance^13^ and E-field-tunable magnetic coupling.^14^ In MFPs, E-fields applied with a scanning tunneling microscope can lower the tautomerization barrier,^10^ yet selective gated control of specific tautomers remains unrealized. One potential limitation is the high symmetry of MFPs, leading to different rotationally equivalent tautomers and low or zero dipole moments, reducing the coupling to external fields.

To explore more asymmetric tautomerism, we consider metal-free benziporphyrins (MFBPs), in which one five-membered pyrrole ring is replaced by a six-membered π-conjugated ring.^15,16^ This substitution introduces a slight out-of-plane distortion and, when suitably functionalized, provides an additional site for proton transfer. As a result, MFBPs can host a broader range of structurally and energetically distinct tautomers. We show that these increased degrees of freedom create a versatile platform for highly tunable, E-field-responsive molecular devices.

We employ density functional theory (DFT) and quantum transport calculations to investigate an experimentally synthesized MFBP, 22-hydroxybenziporphyrin,^17^ which incorporates a six-membered phenol fragment and exhibits tautomerism. This six-membered ring lowers the symmetry of the molecule compared to the tetrapyrrole macrocycle structure of MFPs that, in turn, leads to three distinct low-energy tautomers. The two most stable tautomers, **T1** and **T2**, rapidly interconvert in solution at room temperature, while a slightly higher-energy tautomer, **T3**, likely serves as an intermediate in this interconversion.^17,18^ Despite their subtle chemical differences, these tautomers show markedly different electronic structures. **T1** and **T3** are antiaromatic around the macrocycle, whereas **T2** exhibits local aromaticity around the phenol fragment (see Figure 1).

**Figure 1:**
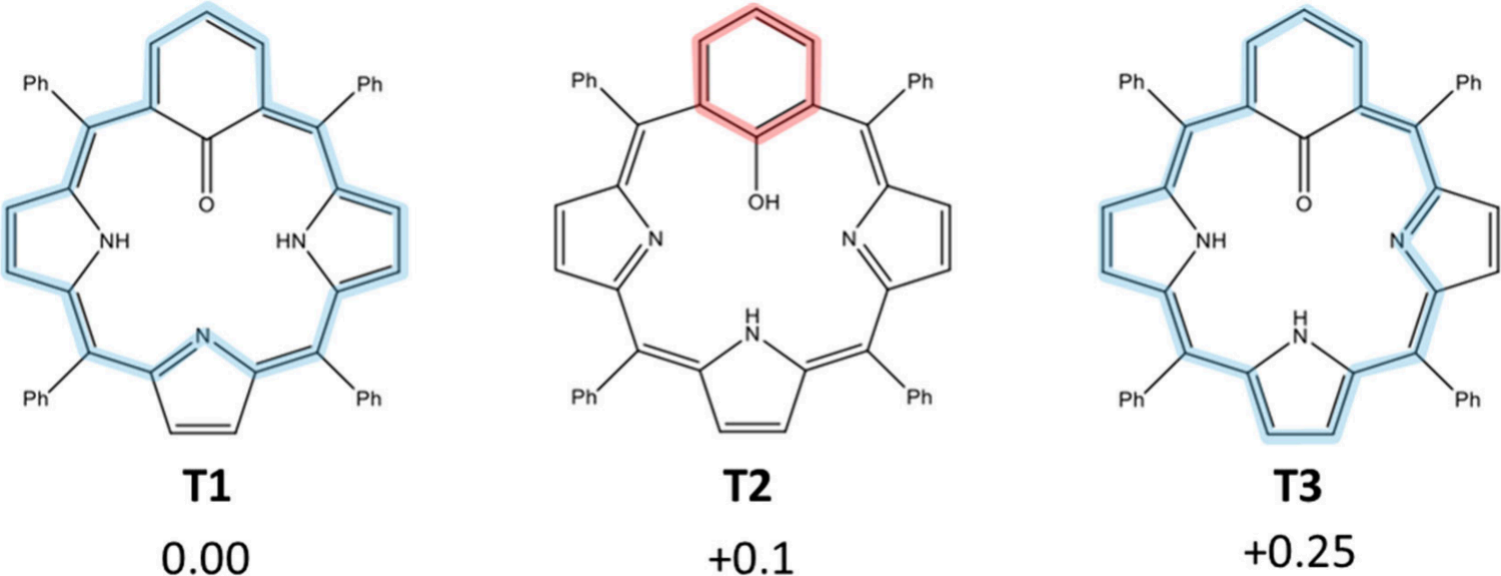
Chemical structures of the three considered MFBP tautomers (**T1**–**T3**). Relative calculated total energies for the zero applied E-field are given in electronvolts with respect to **T1**. The antiaromatic 20-π-electron pathways around the macrocycles are highlighted in blue in **T1** and **T3**. The local six-π-electron aromatic pathway around the phenol ring is highlighted in red in **T2**.

Porphyrinoids have long been studied as systems in which aromaticity can be tuned via structural modification or redox chemistry.^19^ In such systems, topology- or redox-induced switching between aromatic and antiaromatic states can strongly influence molecular conductance.^20,21^ In the MFBP studied here, electron delocalization and aromaticity are intrinsically controlled by tautomerism, offering a novel mechanism for modulating the electronic structure. We theoretically explore how externally applied E-fields can selectively stabilize specific tautomers and how these tautomer-dependent states produce pronounced changes in conductance.

We demonstrate, for the first time, that experimentally realizable, suitably oriented E-fields can directly gate tautomerism in MFBPs. This gating can selectively stabilize each of the three tautomers, thereby controlling the MFBP aromaticity. This E-field-driven switching is independent of any bias current, distinguishing it from previously reported conductance-controlled tautomerism. We then investigate single-molecule junctions for the three tautomers as well as junctions formed by two fused MFBPs with all six possible tautomeric combinations. In all cases, we observe one or two stepwise jumps in the current as the bias voltage increases. The magnitude and voltage dependence of these jumps are specific to each junction, providing distinct, measurable transport signatures that reflect the underlying tautomeric and aromatic states. Notably, in single MFBP junctions, the predicted on/off ratio at low bias can reach 500, reflecting a strong switching response arising from the interchange between aromatic and antiaromatic tautomers. By modifying the chemical linkers between the MFBPs and the junction contacts, this discrete multistate behavior can also be transformed into a more gradual current–voltage response, characteristic of a molecular wire, accompanied by enhanced coherent transport.

Taken together, these results highlight the versatility of MFBPs as E-field-responsive units, capable of functioning both as multistate molecular switches and as tunable molecular wires. Overall, MFBPs offer an enriched range of electronic and transport properties, providing new opportunities to expand the functionality of porphyrinoid-based molecular devices.

## Effect of Applied E-Field on Tautomer Energetics

Figure 2 shows how each tautomer’s fractional population varies with applied E-field strength (0–0.9 V/Å) for two in-plane E-field directions at 298.15 K. These populations are based on the calculated field-induced relative energetics of each tautomer and should not be interpreted as a measure of kinetic accessibility. In each case, the applied E-field is parallel to the plane of the three nitrogen atoms of the respective tautomer with an angle referenced to the connecting axis of the junction, which is set to 0°. Details of the DFT calculation carried out with the Gaussian09 ^22^ code can be found in the Supporting Information (SI). We note that the examined E-field strengths can be achieved with devices based on dual ionic liquid gating or STM tips, which can generate E-fields up to 0.4 V/Å^23^ or 2 V/Å,^24^ respectively.

**Figure 2:**
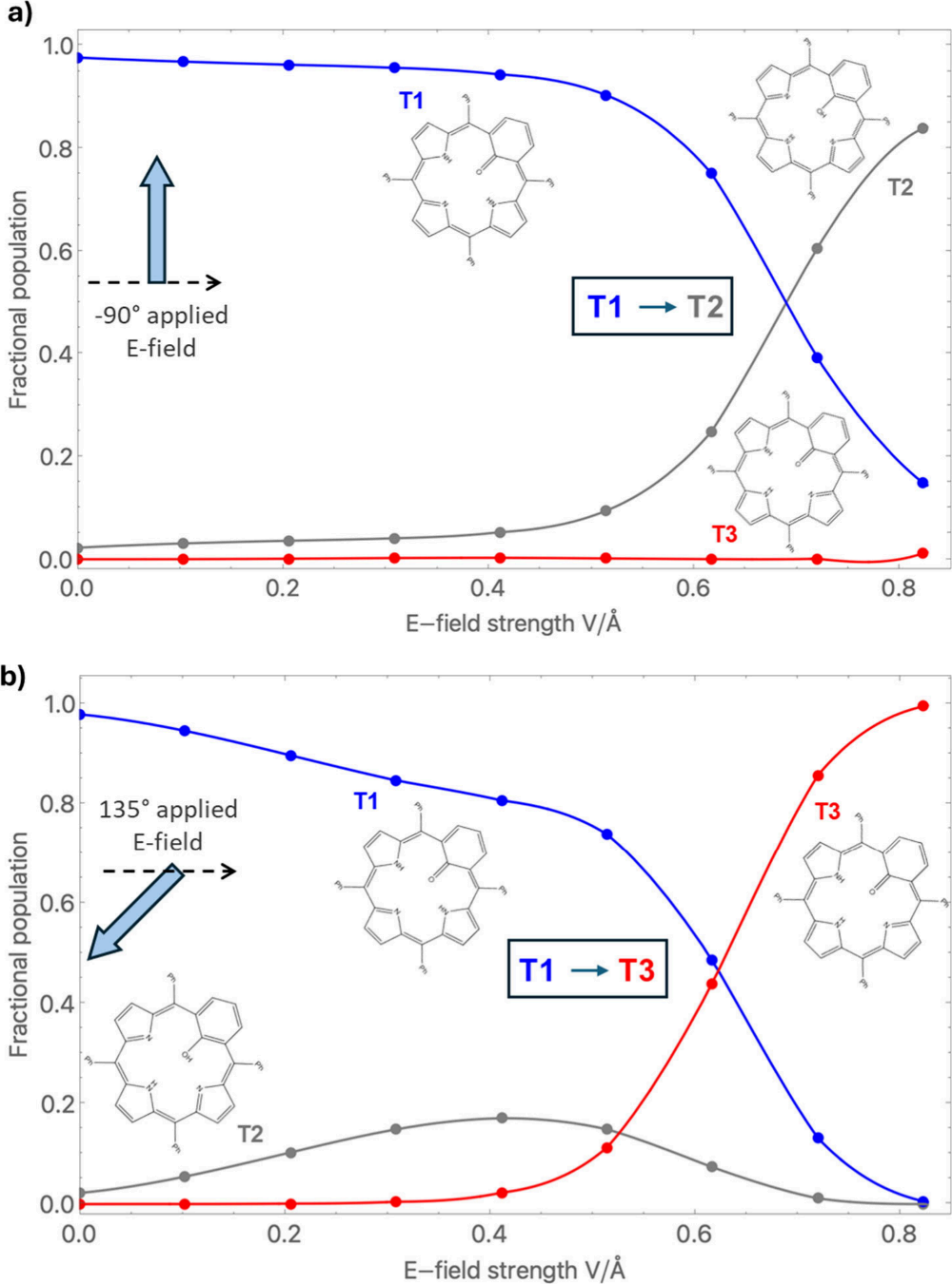
Fractional populations for each tautomer at 298.15 K with respect to the applied E-field strength for field directions of (a) −90° and (b) 135°. Note that the field directions are given relative to the connection axis used in the molecular junctions (see dashed arrows). The inset structures of each MFBP tautomer are also rotated so that they are aligned with the same connection axis.

For low E-field strengths for both considered field directions, **T1** is the dominant tautomer, as expected from its relatively high zero-field stability (see Figure 1). For a −90° applied E-field (i.e., perpendicular to the junction axis), the competition is mainly between **T1** and **T2**. With increasing field strength, the fraction of **T2** slowly rises. This persists up to ∼0.4 V/Å, above which the population of **T2** starts to increase more rapidly with respect to changes in E-field strength, while that of **T1** decreases correspondingly. At an E-field strength of ∼0.69 V/Å, the fractional weight of these two tautomers interchanges. This **T1** → **T2** crossover is reinforced with further increases in E-field strength. These tendencies are plotted in Figure 2a.

For the 135° E-field direction (see Figure 2b), we observe a small gradual decrease in the dominance of **T1** with an increase in field strength up to 0.4 V/Å due to a small increase in the fraction of **T2**. For E-fields above 0.4 V/Å, the prevalence of **T2** falls away while **T3** starts to strongly compete with **T1**. At an E-field strength of ∼0.63 V/Å, there is a crossover between the fractional populations of **T1** and **T3** such that **T3** becomes the dominant tautomer. Further increases in E-field strength leads to a greater stabilisation of **T3** relative to **T1**.

For all tautomers, the applied E-field causes only very minor structural distortions to the MFBP skeleton, indicating that the E-field-induced proton transfer would be a relatively efficient process. Furthermore, the observed E-field-induced tautomer stabilities can be qualitatively rationalized by the relative energies of the induced dipoles of each tautomer in each applied E-field scenario (see the SI).

## Quantum Transport through Single MFBP Junctions

We first consider transport through junctions based on single tautomers (**J**_**T**__***n***_) connected to two gold electrodes via two opposing pendant aryl rings (see the example in Figure 3a). The details of the transport calculations performed with the ARTAIOS code^25^ can be found in the SI. As these calculations were performed without explicitly applied E-fields, shifts of orbital energies through the Stark effect are not accounted for. Orbital energies of the isolated tautomers under E-fields indicate Stark shifts of up to ∼1 eV at the strongest gating fields considered. This effect would lead to a corresponding shift in the required bias voltage for transmission. For the sake of simplicity, we assume that the gating field is applied in a pulse-like manner to induce the necessary tautomerism but is then removed for measuring the conductance response. This mode of operation relies on the persistence of an E-field-induced tautomeric conversion after the pulse is applied, which will be discussed in the next subsection. We note that the E-field induced by the bias voltage along the junction axis is approximately two orders of magnitude lower than a typical gating field (as estimated by assuming a linear voltage drop of 0.1 V over the junction length of 20 Å). This is likely to be an upper bound estimate as the main voltage drops are typically at the electrode–molecule contacts. The corresponding Stark shifts are much smaller (<0.05 eV) and thus would cause only very minor changes to the obtained current versus voltage characteristics.

**Figure 3:**
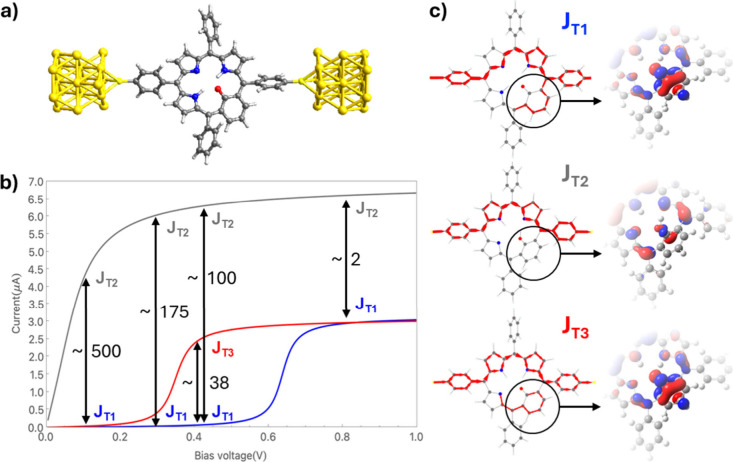
(a) Structure of the molecular junction model with the T1 tautomer linked to two gold electrodes via sulfur atoms (**J**_**T1**_). Atom color key: C - dark gray, O - red, N - blue, H - light gray, S - light yellow, Au - dark yellow. (b) Calculated current with respect to bias voltage for three single MFBP junctions for junctions: **J**_**T1**_ (blue line), **J**_**T2**_ (gray line), and **J**_**T3**_ (red line). Reversible arrows indicate possible E-field-induced tautomeric population shifts following Figure 2. Numbers denote approximate on/off current ratios at specific bias voltages. (c) Local transport pathways at maximal transmission (left). The participation of the associated HOMO orbitals around the macrocyclic six-membered ring in each tautomer is shown to the right.

In Figure 3b, we report the calculated current versus bias voltage curves for each tautomer, each showing a single step-like increase in current at a specific bias voltage that then plateaus afterward. Double-headed arrows indicate the tautomeric switching that would be necessary to induce shifts between these current regimes for selected bias voltages. For device use, E-field switching between tautomers should yield a large current change at a fixed bias, with the on/off ratio measuring the switching efficiency. For bias voltages between 0 and 0.1 V, the current through **J**_**T1**_ stays below 1 nA whereas that through **J**_**T2**_ increases rapidly to more than 3.5 μA. Switching between **T1** (off) and **T2** (on) via an applied E-field (Figure 3a) would therefore yield an on/off ratio of ∼500 across this bias range. For progressively larger bias voltages, this on/off ratio decreases but remains above 100 up to a bias voltage of 0.4 V. These absolute currents are expected to be larger than in typical experimental single-molecule measurements due to the idealized contact coupling, the absence of inelastic scattering, and the coherent transport assumed in the NEGF calculations. However, the on/off ratios rather than the absolute currents provide a more robust measure of the intrinsic switching contrast of the MFBP junctions. We note that the finite bias on/off ratio associated with **T2**/**T1** tautomerism is more than an order of magnitude higher than that calculated^9^ or measured^11^ for a similar tautomerism based conductance changes in symmetric MFPs.

For the case of **T2** ↔ **T3** tautomerism, both MFBP tautomers have similar *I* versus *V* characteristics for many of the bias voltages sampled. The currents appreciably diverge for an approximate bias voltage range of 0.3–0.6 V, where switching is thus possible, with a maximum on/off ratio of ∼38 at a bias voltage of ∼0.4 V. At this bias voltage, switching to **T2** is also possible with an on/off ratio of ∼100. In principle, this could permit three-state switching (**T2** ↔ **T1** ↔ **T3**) with a bidirectional gating field.

Local current analysis (Figure 3b) reveals that **J**_**T2**_ exhibits the most direct conduction pathway, which proceeds predominantly along a single conjugated route connecting the electrodes, reducing scattering and destructive interference, in line with its relatively high conductance. In contrast, the more bifurcated current pathways in **J**_**T1**_ and **J**_**T3**_ display lower overall conductance, reflecting the impact of competing routes and partial current cancellation within the molecular framework. These tendencies are likely linked to the local aromaticity of the six-membered ring of **T2** versus the extended macrocycle antiaromaticity of **T1** and **T3** (see Figure 1). Antiaromatic systems are known to feature high-lying, strongly interacting frontier orbitals that facilitate improved junction performance^26,27^ and efficient electronic coupling.^28^ Conversely, the stabilizing effect of aromaticity tends to suppress these antiaromaticity-enabled channels. Here, indeed we observe that the local aromaticity of the phenol fragment in **T2** (Figure 1b) hinders transport through it. However, this also leads the transport to take a more direct (nonbranching) path through the MFBP molecule. Conversely, when the six-membered ring is part of an antiaromatic macrocycle (i.e., **T1** and **T3** (Figure 1a,c)), an increase in branching currents through the ring is permitted, leading to an overall decrease in direct source-to-drain transport.

The bias voltages at which junction-specific current increases correspond to the maximum transmission energy. In our junctions, this corresponds to the energy of the highest occupied molecular orbital (HOMO) of the system in each case. This is consistent with a transport regime dominated by coherent molecule-centered transport. The main difference between the HOMOs of these tautomers is the relative involvement of the six-membered ring. In **T1** and **T3**, the p orbitals of the ring’s carbon atoms (particularly those bonded to the methine bridges and the carbon atom attached to oxygen) make substantial contributions to the HOMO. In contrast, **T2** exhibits an only minor contribution from the carbon atom bonded to oxygen, leading to negligible overlap between the ring and macrocycle π-systems. The limited orbital participation of the six-membered ring in **T2** distinguishes it from those of **T1** and **T3** (see Figure 3c). Although there is not a simple relation between orbital participation and local transport, these orbital differences are consistent with the observed conductance trends. We note that the orbitals contributing to the aromatic participation of the six-membered ring in **T2** are highly stabilized with respect to the HOMO (by ∼1.3 eV (see the SI)) and are thus not available for transport at low bias voltages. Due to the large stabilization energy, this aromatic suppression of branching currents is expected to be relatively robust with respect to moderate thermally induced structural fluctuations.

## Tautomerism Barriers

Beyond the thermodynamic picture, we also calculated the 0 K tautomerization barriers at zero field and under a 0.8 V/Å E-field. Figure 4a shows the resulting energy landscapes for the three tautomers with an applied E-field in the −90° direction (Figure 2b). Considering the E-field to be applied as a finite pulse to drive tautomerism, we can then follow subsequent relative time scales related to the associated conductance changes.

**Figure 4:**
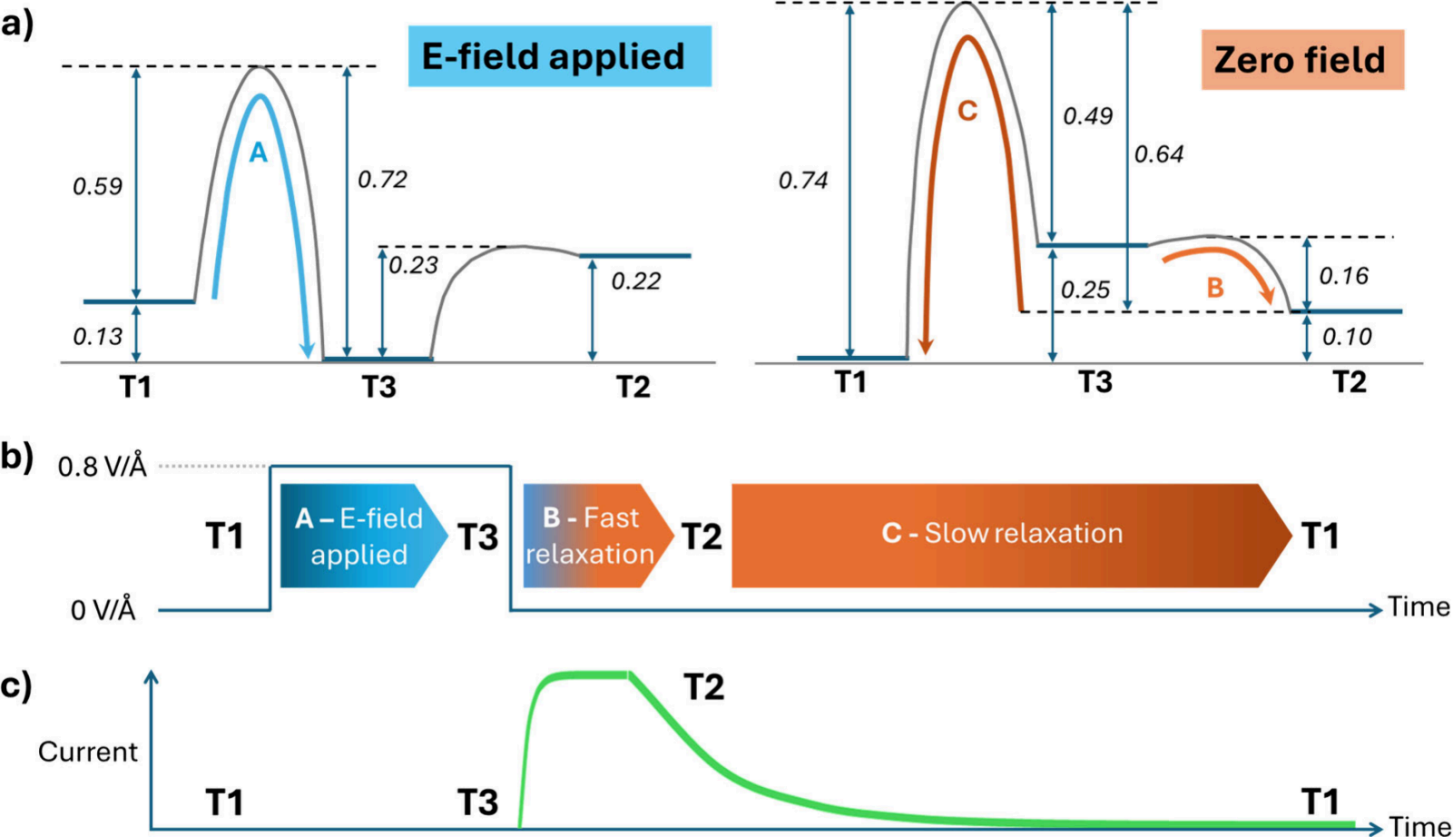
(a) Energy landscape of all three tautomers and tautomerism barriers with and without an applied E-field of 0.8 V/Å. (b) Schematic time line of relative tautomerism processes upon application and removal of an E-field pulse. (c) Schematic time line of the junction current upon application and removal of an E-field pulse.

The zero-field scenario reveals a relatively large barrier of 0.74 eV going from **T1** to **T3**, with a negligible barrier from **T3** to **T2**. **T3** thus acts as a short-lived intermediate in the **T1** → **T2** transition. From experiment, tautomerism between **T1** and **T2** readily occurs in solution at room temperature, with **T3** identified as a likely intermediate. The experimentally derived activation energy for proton transfer in MFBP is estimated to be 0.33 eV.^17^ We note that our barrier is based on energetics at 0 K (i.e., not a finite temperature free energy barrier) and does not include the influence of solvent, both likely contributing to its relatively higher calculated magnitude. Assuming that our calculated values approximate those for a solvent-free junction, the large zero-field **T1** → **T3** barrier means that tautomerism from the thermodynamically preferred **T1** state would be strongly suppressed at room temperature and zero field.

With an applied E-field, the thermodynamic driving force moves toward **T3** and the **T1** → **T3** barrier is significantly reduced to 0.59 eV. This modest tautomerism barrier would be typically crossed on a millisecond time scale at room temperature. This barrier could be overcome more easily by increasing temperature or employing current bursts. Once **T3** is reached, **T2** is easier to access than **T1** and a **T3**/**T2** dynamic equilibration would be quickly established with **T3** being dominant. When the E-field is removed, the energy landscape reverts to its zero-field topology. Here, the **T3** → **T2** channel becomes effectively barrierless and **T2** now becomes dominant in the **T3**/**T2** dynamic equilibrium, yielding a large, step-like increase in junction current. However, eventually, **T2** will revert back to **T1** due to thermodynamics. The **T2** → **T1** pathway involves two proton transfers. The barrier of the first step (0.16 eV) is significantly lower than that of the second step (0.49 eV). Accordingly, the kinetics of the **T2** → **T1** process has a rate-determining step (**T3** → **T1**) preceded by the fast and reversible **T2** → **T3** step, which reaches equilibrium. In such a scenario, the effective **T2** → **T1** barrier is given by the sum of the energy barrier of the slow step and the energy difference between **T3** and **T2** (i.e., 0.49 eV + 0.15 eV = 0.64 eV). This effective barrier then dictates the slower relaxation of the system back to **T1** with a progressively decreasing current. Because the zero-field **T2** → **T1** barrier is higher than the field-induced **T1** → **T3** barrier, the **T2** → **T1** rate is about an order of magnitude slower than the **T1** → **T3** rate at room temperature. The separation between these time scales could also be further increased by cooling. Panels b and c of Figure 4 schematically illustrate the E-field pulse effects on tautomerism and current.

## Transport in Fused Tautomer Junctions

To further assess the potential of MFBPs as multistate molecular switches, we calculated the transport characteristics of junctions formed from all six possible combinations of two fused MFBP tautomers (denoted as **J**_**T*n*‑T*m***_). The MFBPs are fused via two pyrrole rings in each tautomer, following the triply linked connectivity commonly employed to construct highly conjugated porphyrin-based molecular wires.^5^ Such edge-fused porphyrin architectures, often termed porphyrin tapes, have been extensively investigated for metal-containing porphyrins, while hybrid tapes incorporating MFPs have also been experimentally realized.^29^

Because the six-membered benzenoid ring lowers the symmetry of the MFBP macrocycle compared with tetrapyrrole MFPs, fusion through pyrrole rings requires a relative rotation of the two MFBP units. In all fused junctions considered here, one MFBP is rotated by 90° with respect to the other (see the **J**_**T1‑T1**_ junction in Figure 5a); the corresponding structural models for the remaining junctions are provided in the SI.

**Figure 5:**
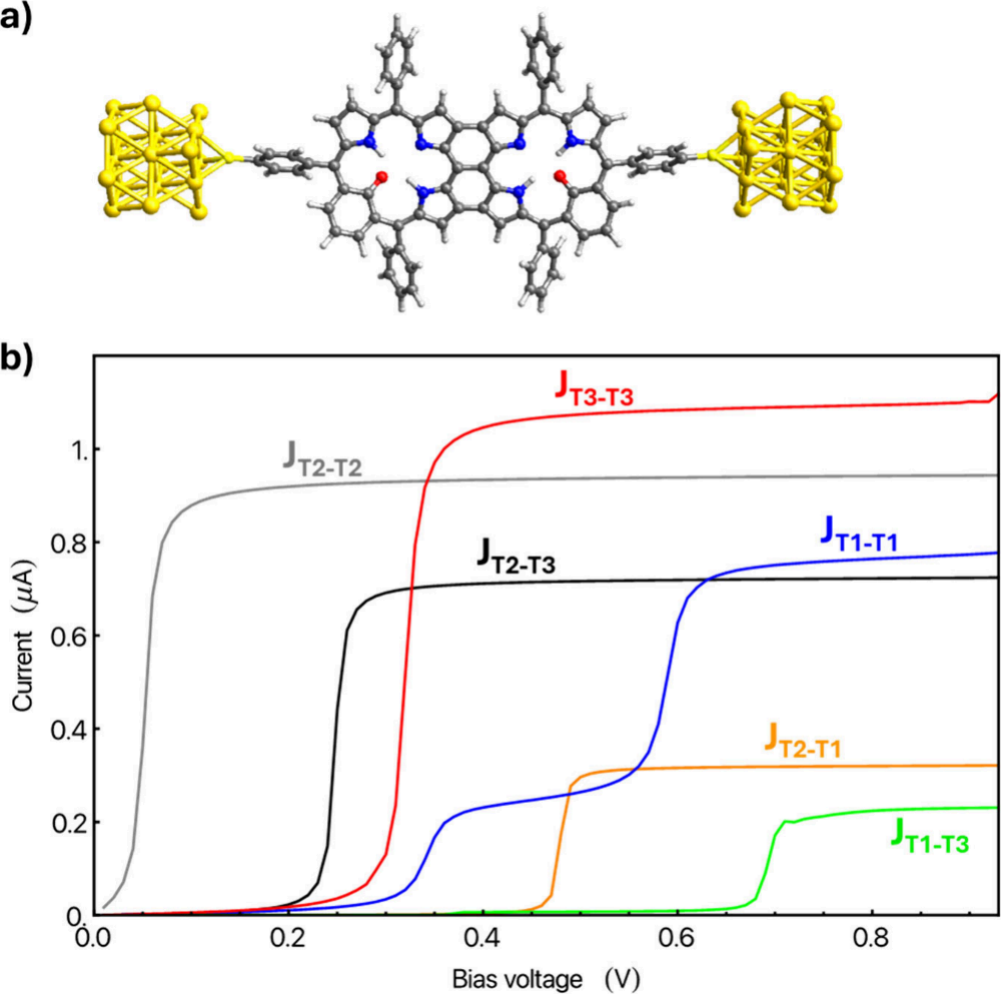
(a) Structure of the molecular junction model with two fused **T1** MFBP tautomers linked to two gold electrodes via sulfur atoms (**J**_**T1‑T1**_). The atom color key follows that used in Figure 3. (b) Calculated current with respect to bias voltage for six junctions comprised of different fused combinations of two tautomers.

Similar to the single-tautomer junctions, the fused systems generally exhibit a single stepwise increase in current at a characteristic bias voltage within the range studied, except for the **J**_**T1‑T1**_ junction, which displays a more complex two-step current–voltage response (see Figure 5b). This response is due to the emergence of two accessible transmission peaks in the bias voltage range considered in this case (see the SI). Overall, junctions composed of identical MFBP tautomers yield the three highest maximum currents. However, in all cases, the fused junctions exhibit significantly reduced current compared to their single-tautomer counterparts.

In line with the results of the single MFBP junctions, most of the fused junctions exhibit branching currents, which are generally detrimental to transport. The transport path in **J**_**T2‑T2**_ closely matches that of **J**_**T2**_, with the aromatic six-membered rings again suppressing branching currents (see the SI). We note that, unlike in the single-MFBP junctions, **J**_**T3‑T3**_ has a slightly higher maximum current than **J**_**T2‑T2**_, which is difficult to rationalize from the local transport paths.

Importantly, all six fused junctions display stepwise current increases at distinct bias voltages and reach different maximum current levels, providing six clearly distinguishable transport signatures. To date, experiments have achieved four-state tautomer-based conductance-based switching (i.e., not gate-induced) at high bias voltages and low temperatures for single-MFP junctions.^11^ Although the E-fields required to access these different tautomeric combinations are probably more complex than those for single tautomers, these results underscore the potential of MFBP-based junctions as multistate molecular electronic elements. Unlike conventional two-state on/off molecular switches, these systems offer multiple stable, addressable configurations, potentially enabling more complex switching schemes, increased information density, and enhanced functionality for molecular-scale logic and memory applications.

## MFBP-Based Molecular Wires

Unlike switchable molecular junctions, which exhibit stepwise *I–V* characteristics due to discrete changes in conductance, molecular wire-type junctions typically display relatively high currents that vary smoothly with applied bias, reflecting continuous electron transport through a conjugated pathway. It is well-known that the nature of the molecule–electrode connection in porphyrin junctions can significantly affect the observed transport characteristics.^30^ Here, we examine these effects in three different variants of **J**_**T1**_ (see Figure 6a), termed **J**_**T**__**1a**_–**J**_**T1c**_. Case **J**_**T**__**1c**_ is the junction shown in Figure 3a. In **J**_**T1b**_, we replace the aryl rings linking the molecule with the electrodes with acetylenic linkers. In **J**_**T1a**_, we further remove the two pendant aryl rings of the MFBP.

**Figure 6:**
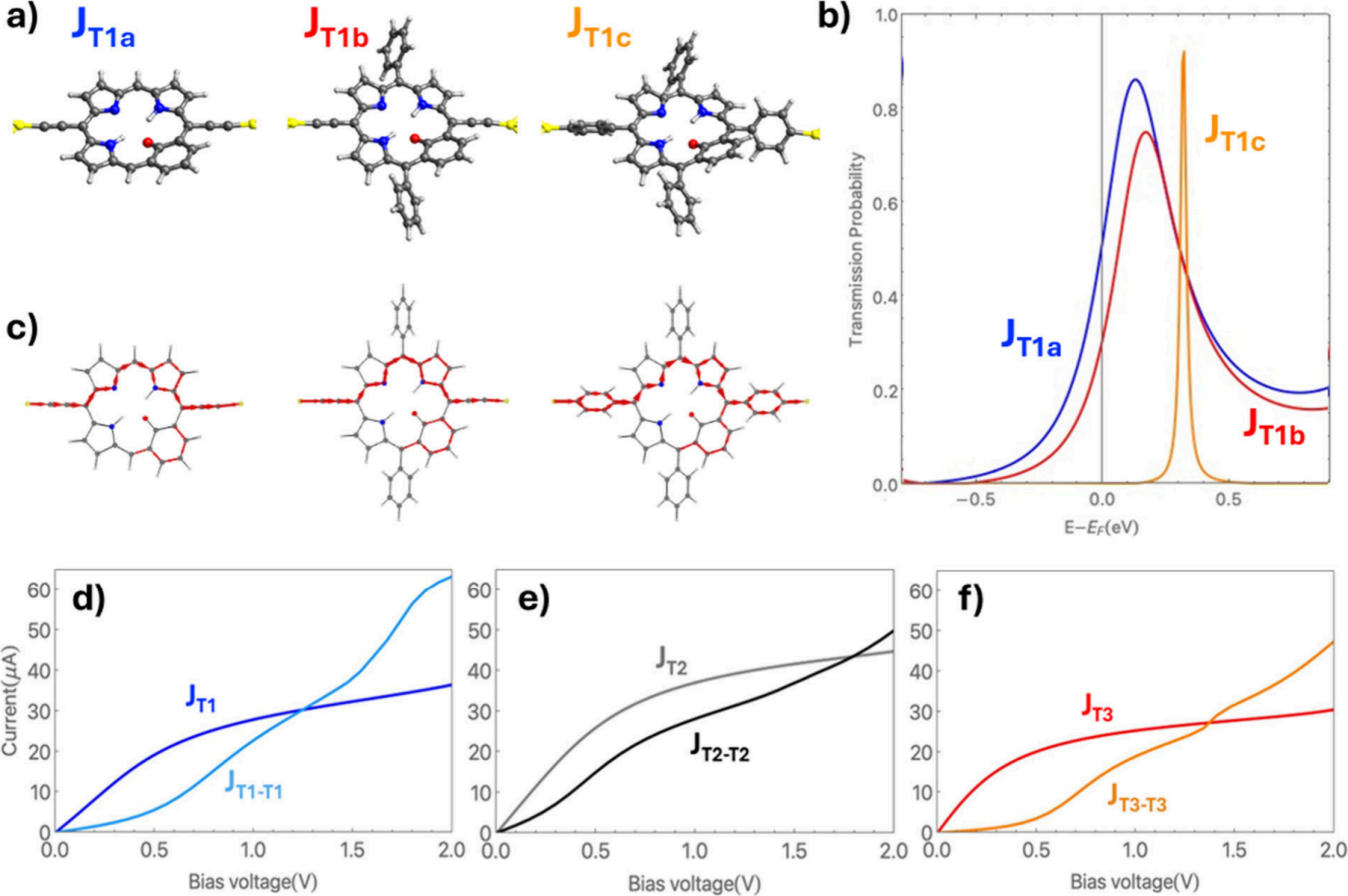
(a) Structures of three **J**_**T1**_ variants: **J**_**T1a**_, no aryl ring decoration and acetylenic electrode linkers; **J**_**T1b**_, with aryl ring decoration and acetylenic electrode linkers; **J**_**T1c**_, aryl ring decoration and electrode linkers. Note that **J**_**T1c**_ is equivalent to **J**_**T1**_ in Figure 3. The atom color key follows that in Figure 3. (b) Transmission functions vs energy deviation from the Fermi level of the electrodes and (c) local transport currents corresponding to the maximum transmission in panel b. Comparison of current vs bias voltage characteristics for single MFBP and wire-like fused MFBP-based junctions with acetylenic electrode linkers and no pendant aryl ring decoration: (d) **J**_**T1**_ and **J**_**T1‑T1**_, (e) **J**_**T2**_ and **J**_**T2‑T2**_, and (f) **J**_**T2**_ and **J**_**T3‑T3**_.

**J**_**T**__***1c***_ exhibits a narrow transmission peak at ∼0.3 eV (see Figure 6b), which corresponds to the single stepped increase in current at ∼0.6 V shown in Figure 3 (see eq 4 in the SI). This result is in line with the known effect of aryl rings to selectively tune the degree of conjugated π-orbital overlap via their twist angle when used as linkers in junctions^31^ and materials.^32^ Conversely, **J**_**T**__**1a**_ and **J**_**T**__**1b**_ display significant broadening and shifting of the transmission function peaks. Acetylenic linkers provide rigid, linear, and highly conjugated connections between molecular backbones and electrodes, leading to strong molecule–electrode coupling and significant orbital hybridization. As a result, transmission resonances are substantially broadened compared with junctions containing more weakly coupled twisted aryl linkers. We note that the local transport pathways in each case (Figure 6c) are relatively unaffected by the nature of the connection of the MFBP to the electrodes, in which we employ acetylenic linkers to the gold electrodes and no aryl ring decorations. As shown in Figure 6d–f, the acetylenic-linked single-molecule junctions exhibit currents that are at least an order of magnitude higher than those of in the aryl-linked junctions (Figure 3b).

As minimal models of MFBP molecular wires, we consider variants of **J**_**T1‑T1**_, **J**_**T2‑T2**_, and **J**_**T3‑T3**_, in which we employ acetylenic linkers to the gold electrodes and no pendant aryl ring decorations. The edge-fused MFBP wires always show a more complex pattern of increasing current with respect to bias voltage as compared to the single-molecule junctions. While the current in the single-molecule systems tends to start leveling off at around 0.5 V, the current in the wire-like systems keeps increasing and eventually surpasses that of the former for bias voltages above approximately 1.5 V. This behavior is quite unlike that of the aryl-linked fused MFBP junctions reported in Figure 5, which have maximal currents that are significantly lower than the corresponding single-MFBP junctions. Recent theoretical and experimental studies have demonstrated that edge-fused wires of metal-containing porphyrins and MFPs exhibit increasing conductance with increasing length.^33,34^ This unintuitive phenomenon is due to the rapidly narrowing HOMO–LUMO gap as the length of the wire increases, countering the effect of longer tunneling distances. Our tentative results suggest that a similar effect could also occur in edge-fused MFBP molecular wires.

## Conclusions

Our results demonstrate that E-fields can effectively bias tautomerization in MFBPs. Experimentally achievable E-fields can selectively stabilize three distinct tautomers, each associated with a unique aromatic or antiaromatic character and conductance signatures, providing a clear route for state detection. This direct coupling between tautomerism and aromaticity enables single MFBPs to function as three-state molecular switches with high on/off ratios. Extending this concept, fused MFBPs offer multiple tautomeric configurations with distinct conductance profiles, creating opportunities for potential multistate molecular logic and memory elements. By tuning the electrode linkers, these systems can also exhibit wire-like transport with enhanced currents, suggesting a pathway toward MFBP-based molecular interconnects. Taken together, these findings position MFBPs as a versatile and scalable platform for E-field-responsive molecular electronics, bridging fundamental tautomeric control with practical device architecture potential.

## Supplementary Material



## Notes

Comparison of MFBP tautomer relative energies with respect to applied E-field, structural models of fused MFBP junctions, orbitals associated with aromaticity of the six-membered ring in T2, and local transport paths and transmission peaks for fused MFBP junctions (PDF)
