# Supplementary material for: Electric-Field-Induced Tautomerism in Metal-Free Benziporphyrins Enables Aromaticity-Controlled Conductance Switching
Source: Nano Lett. 2026 Apr 15;26(16):5396–403. doi: 10.1021/acs.nanolett.5c06447 (PMC13133910; doi:10.1021/acs.nanolett.5c06447)
Supplement: Supplementary file 1 [file nl5c06447_si_001.pdf]

Supporting Information for:

Electric-Field-Induced Tautomerism in Metal-Free Benziporphyrins Enables Aromaticity-Controlled Conductance Switching

Yenni Ortiz-Acero[†1], Arnau Cortés-Llamas[†1], Jordi Ribas-Arino[1], Stefan T. Bromley*[1,2]

[1]*Departament de Ciència de Materials i Química Física & Institut de Química Teòrica i Computacional (IQTCUB), Universitat de Barcelona, c/Martí i Franquès 1-11, 08028 Barcelona, Spain*

[2]*Institució Catalana de Recerca i Estudis Avançats (ICREA), Passeig Lluís Companys 23, 08010 Barcelona, Spain*

† These authors contributed equally to this work
* Corresponding author: s.bromley@ub.edu

Contents

S1. Density functional calculations

S2. Quantum electron transport calculations

S3. Comparison of MFBP tautomers relative energies with respect to applied E-field

S4. Structural models of fused MFBP junctions

S5. Orbitals associated with aromaticity of the six-membered ring in T2

S6. Local transport paths and transmission peaks for fused MFBP junctions

S1. Density functional theory calculations

Relaxed structures and energies of all freestanding molecules (i.e. without gold electrodes) were obtained via density functional theory (DFT) based optimisations using the PBE0 hybrid exchange-correlation functional [1], and a 6-311G(d,p) basis set, as implemented in the Gaussian09 [2] code. Dispersion interactions were also included using Grimme's D3 scheme [3,4]. Applied electric fields (E-fields) were included via the "Field" keyword to define the direction and strength of an applied uniform E-field. In such calculations, a new term accounting for the potential due to the E-field is added to the external potential of the Kohn-Sham Hamiltonian. The Kohn-Sham equations are then solved taking into account the effective potential and thus the relaxation of the electronic density is self-consistently evaluated with influence of the applied E-field. Uniform E-fields were applied to all tautomers in two in-plane directions (i.e. with respect to the plane defined by the three nitrogen atoms of each tautomer) with field strengths ranging between zero and 0.9 V/Å. To demonstrate the influence of the applied E-field on the relative energetic stabilities of the tautomers, we evaluated the fractional populations of each tautomer ($P(n)$) for all considered E-field strengths and directions

$$P(n) = \frac{1}{Z} e^{\frac{-E_n}{k_B T}} \qquad (1)$$

where E_n is the relative total energy of each tautomer n, T is the temperature (taken to be room temperature, 298.15 K), Z is the total partition function for all three tautomers, and k_B is the Boltzmann constant. Transition states between the energy minima associated with each tautomer were calculated using the eigenvector following algorithm [5] using the same DFT set-up. The effect of the applied E-fields on the barrier heights was estimated from single-point DFT calculations using the structures of the respective zero-field transition states with an explicit E-field applied.

S2. Quantum electron transport calculations

Quantum electron transport calculations employed model junctions in which the reported MFBP-based systems were connected between two gold clusters via sulfur atoms. The transport calculations for each junction were performed without an explicitly applied E-field. We employed gold clusters composed of 19 atoms in a face-centered cubic structure cut from the bulk crystal structure. DFT calculations using the B3LYP exchange-correlation functional [6] together with the LANL2DZ basis set were employed to treat this junction region. LANL2DZ combines effective core potentials (ECPs) with double-zeta quality basis functions for the valence electrons of the Au atoms, with an explicit all-

electron basis set for the lighter elements of the MFBPs. This choice allows for a reliable representation of the electronic structure while minimizing artifacts (e.g. ghost transmission) that can arise in transport calculations [7]. The explicitly represented Au contacts were coupled to virtual semi-infinite leads, to allow for the electron transport through the system to be calculated by means of the nonequilibrium Green's function method (NEGF) [8, 9, 10] as implemented in the ARTAIOS software package [11]. In the NEGF calculations the Green's function of the scattering region (i.e. between the electrodes) $G(E)$ is evaluated following equation 2.

$$G(E) = (E\,S - H - \Sigma_S - \Sigma_D)^{-1} \qquad (2)$$

where E represents the electron energy, H denotes the effective single-particle Kohn-Sham Hamiltonian, and S is the overlap matrix obtained from the DFT calculation. The self-energies $\Sigma_{S/D}$ account for the influence of the source and drain reservoirs, which are coupled to the system via the gold clusters to facilitate electron injection and extraction. These reservoirs are modeled using the wideband approximation [12]. With the Green's function, the transmission function can be calculated as

$$T(E) = 4Tr\left[Im(\Sigma_S)GIm(\Sigma_D)G^{\dagger}\right] \qquad (3)$$

as well as the local transmissions between atoms i and j

$$T_{ij}(E\,) = \operatorname{Im}\,(H_{ij}^{*}\;G_{ij}^{n}) \qquad (4)$$

where the correlation function is given by

$$G^{n} = 2G\,\operatorname{Im}(\Sigma_S)G^{\dagger} \qquad (5)$$

The current I is determined by integrating the energy-dependent transmission function T. The range of integration depends on the equilibrium Fermi energy E_F of the electrodes and the applied bias voltage (V).

$$I(V) = \frac{2e}{h}\int_{E_F-\frac{eV}{2}}^{E_F+\frac{eV}{2}} dE\;T(E,V) \qquad (6)$$

where e is the unit electronic charge and h is Planck´s constant.

The local transmission between pairs of atoms i and j is calculated from the imaginary part of the product of the Hamiltonian matrix element H_{ij} and the corresponding element of the correlation function. Summing these atomic-pair contributions reproduces the global transmission, while retaining spatial resolution of current pathways.

S3. Comparison of MFBP tautomers relative energies with respect to applied E-field

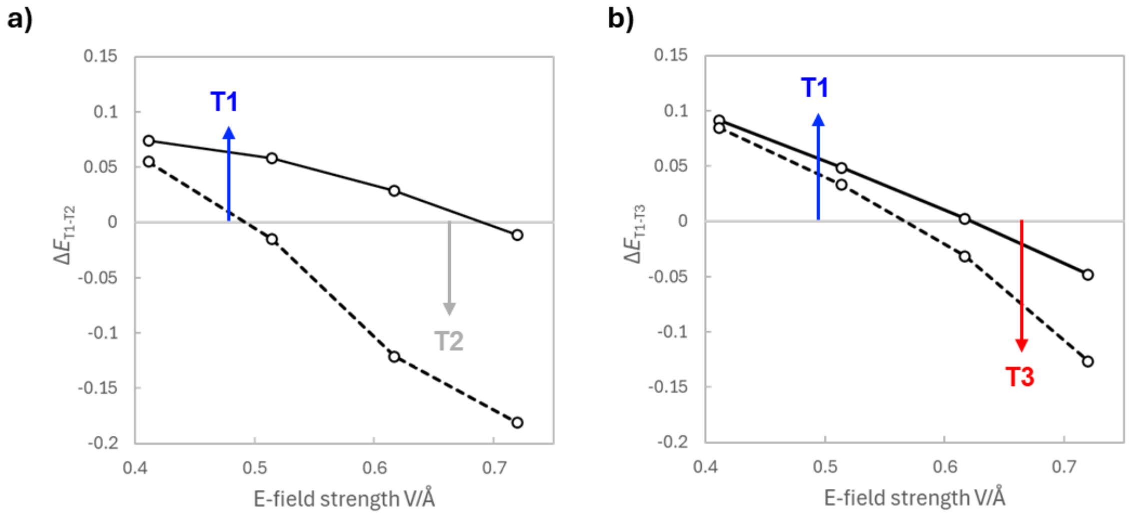

Fig. S1. Comparison of relative total energies of pairs of tautomers with respect to applied E-fields around the crossover E-fields for: a) the **T1** to **T2** transition, and **b)** the **T1** to **T3** transition (see Fig. 2 for field directions in each case). The solid lines correspond to directly calculated energies from the DFT calculations (explicitly including the E-field). Dashed lines correspond to estimates of relative total energies based only on the zero-field gas phase DFT energy differences and the energy of the interaction of the E-field with the calculated total dipole (i.e. fixed + induced) of each tautomer.

S4. Structural models of fused MFBP junctions

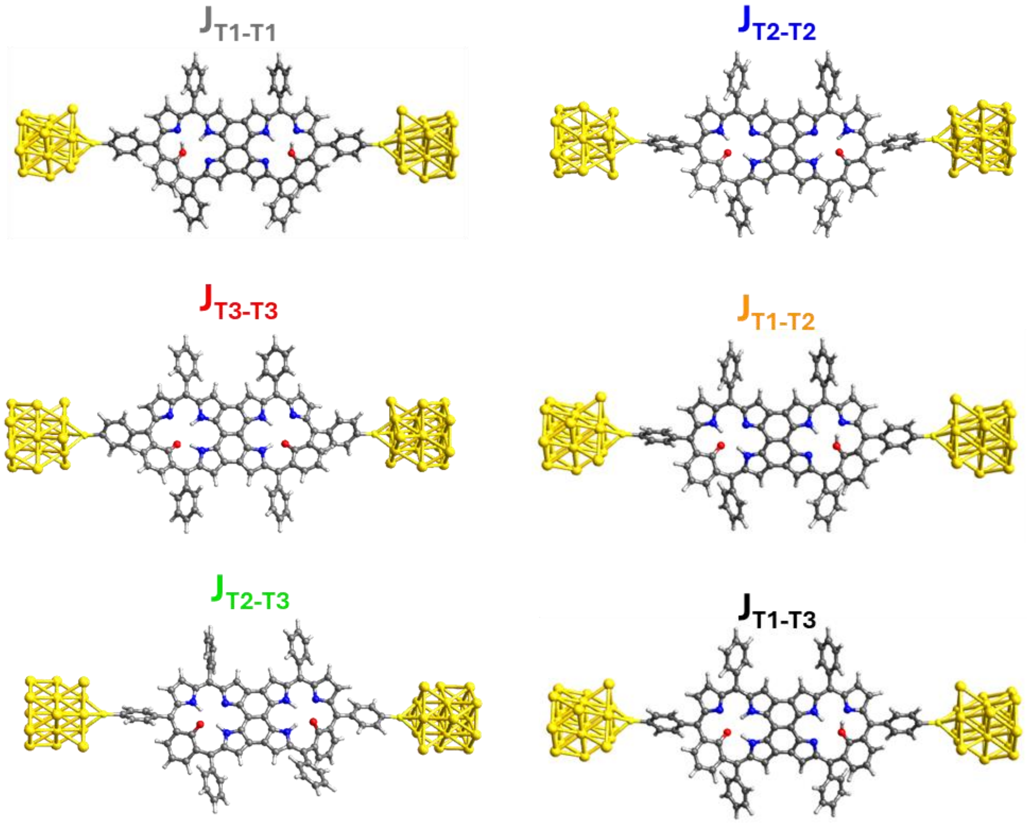

Fig. S2. Structures of molecular junction models ($\mathbf{J_{Tn\text{-}Tm}}$) each comprising different combinations of two fused MFBP tautomers (from: **T1**, **T2**, **T3**) linked to two gold electrodes via sulphur atoms. Atom colour key: C – dark grey, O – red, N - blue, H - light grey, S - light yellow, Au - dark yellow.

S5. Orbitals associated with aromaticity of the six-membered ring in T2

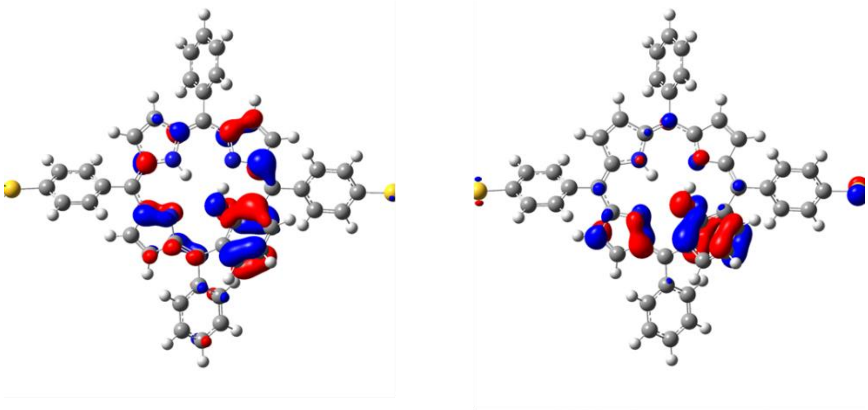

HOMO – 1.27 eV   HOMO – 1.19 eV

Fig. S3. Orbitals and energies (relative to the HOMO) relating to highest aromatic p-orbital participation on the six-membered ring in **T2**.

S6. Local transport paths and transmission peaks for fused MFBP junctions

$J_{T2\text{-}T2}$

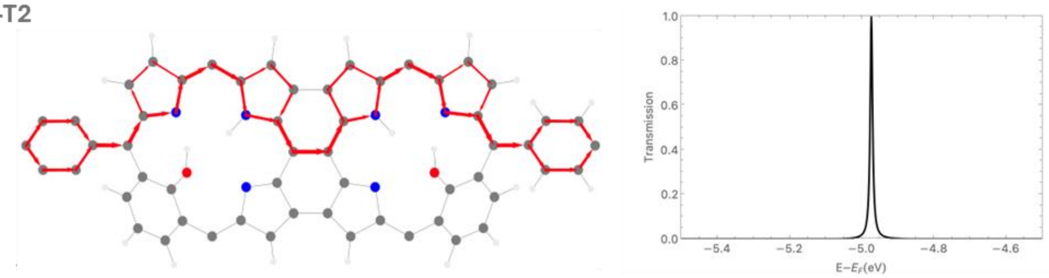

$J_{T1\text{-}T2}$

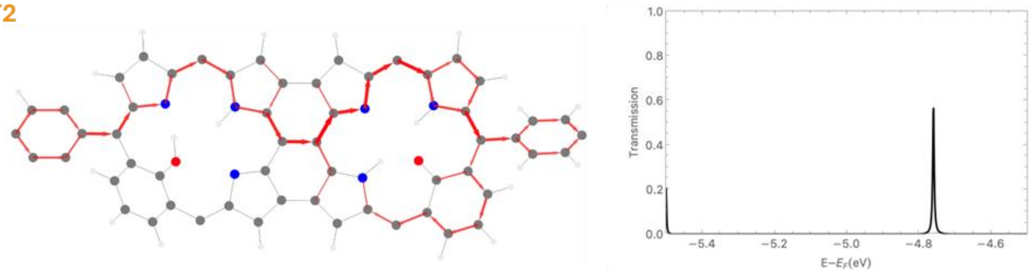

$J_{T2\text{-}T3}$

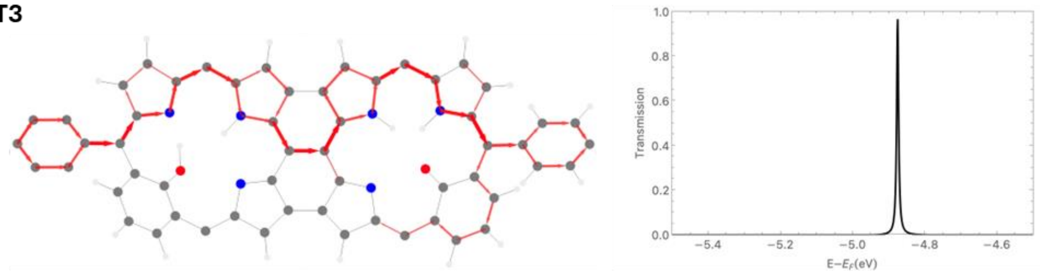

$J_{T1\text{-}T1}$

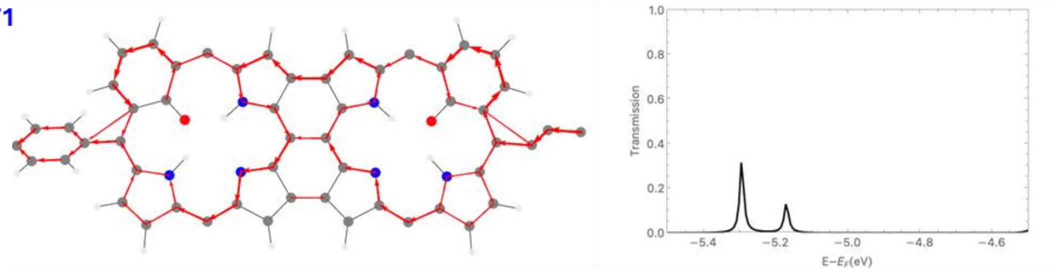

$J_{T1\text{-}T3}$

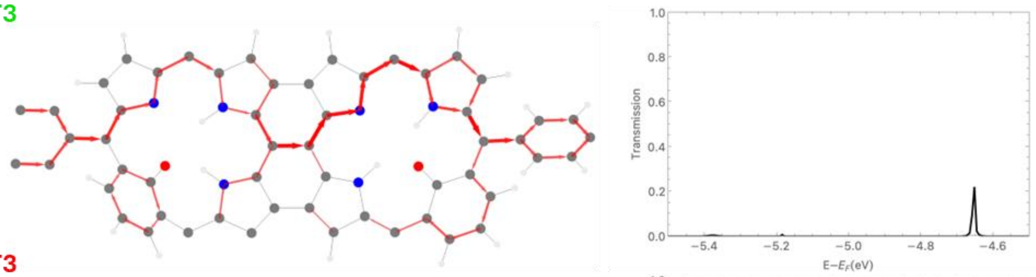

$J_{T3\text{-}T3}$

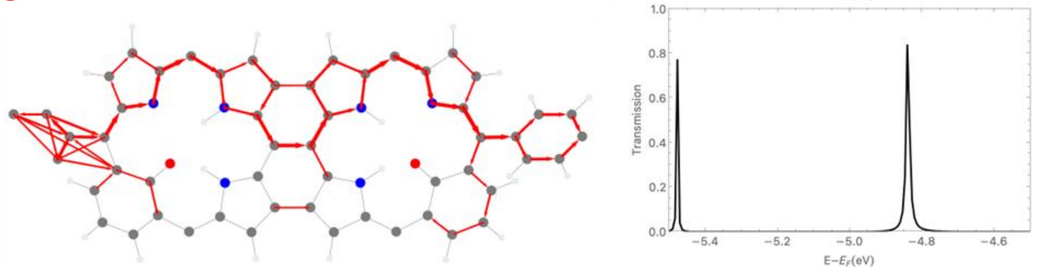

References

[1] Adamo, C.; Barone, V. Toward Reliable Density Functional Methods without Adjustable Parameters: The PBE0 Model. J. Chem. Phys. 1999, 110, 6158–6170.

[2] Frisch, M. J.; et al. Gaussian 09, Revision D.01; Gaussian, Inc.: Wallingford CT, 2013.

[3] Grimme, S.; Antony, J.; Ehrlich, S.; Krieg, H. A Consistent and Accurate Ab Initio Parametrization of Density Functional Dispersion Correction (DFT-D) for the 94 Elements H–Pu. J. Chem. Phys. 2010, 132, 154104.

[4] Grimme, S.; Ehrlich, S.; Goerigk, L. Effect of the Damping Function in Dispersion Corrected Density Functional Theory. J. Comput. Chem. 2011, 32, 1456–1465.

[5] Simons, S.; Jørgensen,P.; Taylor, H.; Ozment, J. Walking on Potential Energy Surfaces. J. Phys. Chem. 1983, 87, 2745-2753.

[6] Stephens, P. J.; Devlin, F. J.; Chabalowski, C. F.; Frisch, M. J. Ab Initio Calculation of Vibrational Absorption and Circular Dichroism Spectra Using Density Functional Force Fields. J. Phys. Chem. 1994, 98, 11623–11627.

[7] Herrmann, C.; Solomon, G. C.; Subotnik, J. E.; Mujica, V.; Ratner, M. A. Ghost Transmission: How Large Basis Sets Can Make Electron Transport Calculations Worse. J. Chem. Phys. 2010, 132, 024103.

[8] Datta, S. Nanoscale Device Modeling: The Green's Function Method. Superlattices Microstruct. 2000, 28 (4).

[9] Camsari, K. Y.; Chowdhury, S.; Datta, S. The Nonequilibrium Green Function (NEGF) Method. In Springer Handbook of Semiconductor Devices; Rudan, M.; Brunetti, R.; Reggiani, S., Eds.; Springer.

[10] Gao, F.; Menchón, R. E.; Garcia-Lekue, A.; Brandbyge, M. Tunable Spin and Conductance in Porphyrin–Graphene Nanoribbon Hybrids. Commun. Phys. 2023, 6, 115.

[11] Deffner, M.; Groß, L.; Steenbock, T.; Voigt, B. A.; Solomon, G. C.; Herrmann, C. ARTAIOS, a Code for Postprocessing Quantum Chemical Electronic Structure Calculations. https://www.chemie.uni-hamburg.de/institute/ac/arbeitsgruppen/herrmann/software/artaios.html.

[12] Verzijl, C. J. O.; Seldenthuis, J. S.; Thijssen, J. M. Applicability of the Wide-Band Limit in DFT-Based Molecular Transport Calculations. J. Chem. Phys. 2013, 138, 094102.
